# Supplementary material for: Degradation of Lignin by Infrared Free Electron Laser
Source: Polymers (Basel). 2022 Jun 14;14(12):2401. doi: 10.3390/polym14122401 (PMC9227113; doi:10.3390/polym14122401)
Supplement: Supplementary file 1 [file polymers-14-02401-s001.zip › polymers-1758434-supplementary.pdf]

Supporting Information

for

Degradation of Lignin by Infrared Free Electron Laser

Takayasu Kawasaki[1]*, Heishun Zen[2], Takeshi Sakai[3], Yoske Sumitomo[4], Kyoko Nogami[3], Ken Hayakawa[3], Toyonari Yaji[5], Toshiaki Ohta[5], Takashi Nagata[2], and Yasushi Hayakawa[3]

[1]Accelerator Laboratory, High Energy Accelerator Research Organization, 1-1 Oho, Tsukuba, Ibaraki 305-0801, Japan.

[2]Institute of Advanced Energy, Kyoto University, Gokasho, Uji, Kyoto 611-0011, Japan.

[3]Laboratory for Electron Beam Research and Application (LEBRA), Institute of Quantum Science, Nihon University, 7-24-1 Narashinodai, Funabashi, Chiba 274-8501, Japan.

[4]Department of Physics, College of Science and Technology, Nihon University, 1-8-14 Kanda Surugadai, Chiyoda-ku, Tokyo 101-8308, Japan.

[5]SR Center, Research Organization of Science and Technology, Ritsumeikan University, 1-1-1 Noji-Higashi, Kusatsu, Shiga 525-8577, Japan.

Correspondence: takayasu.kawasaki@kek.jp

1. IR-FEL spectra at several wavelengths.

IR-FEL spectra tuned to 6.3 μm, 7.1 μm, and 3.0 μm were shown in **Fig. S1**. Each spectrum was obtained by introducing the beam line into the spectrometer. Two mid-infrared FEL spectra at 6.3 μm and 7.1 μm were measured at KU-FEL (Kyoto University), and one near-infrared FEL spectrum at 3.0 μm was measured at LEBRA (Nihon University).

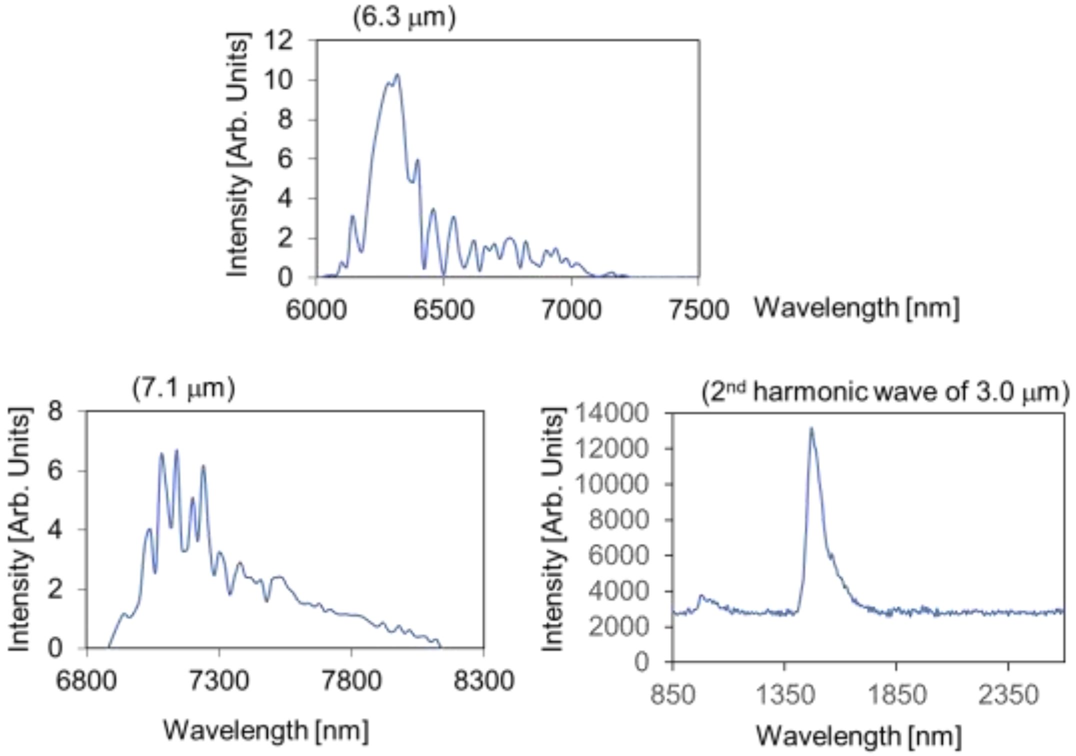

Figure S1. FEL spectrum at 3.0-7.1 μm. The near-IR FEL spectrum was measured as a 2nd harmonic wave (1475 nm).

2. Laser irradiation method

The laser beam was introduced from a beam port to the sample surface by using two reflection mirrors and one parabolic mirror in the case of mid-IR FEL system (**Fig. S2**, upper). In the case of near-IR FEL system (below), the beam direction was controlled by using three reflection mirrors and introduced onto the sample by using a focused lens (CaF_2, f = 100 mm, diameter: 50 2

mm). The lignin powder (about 5 mg) was added in a glass tube, and the IR-FEL was irradiated from the vertical direction at room temperature under atmospheric conditions. The glass tube was sometimes shaken horizontally to give the radiation energy to the whole sample uniformly.

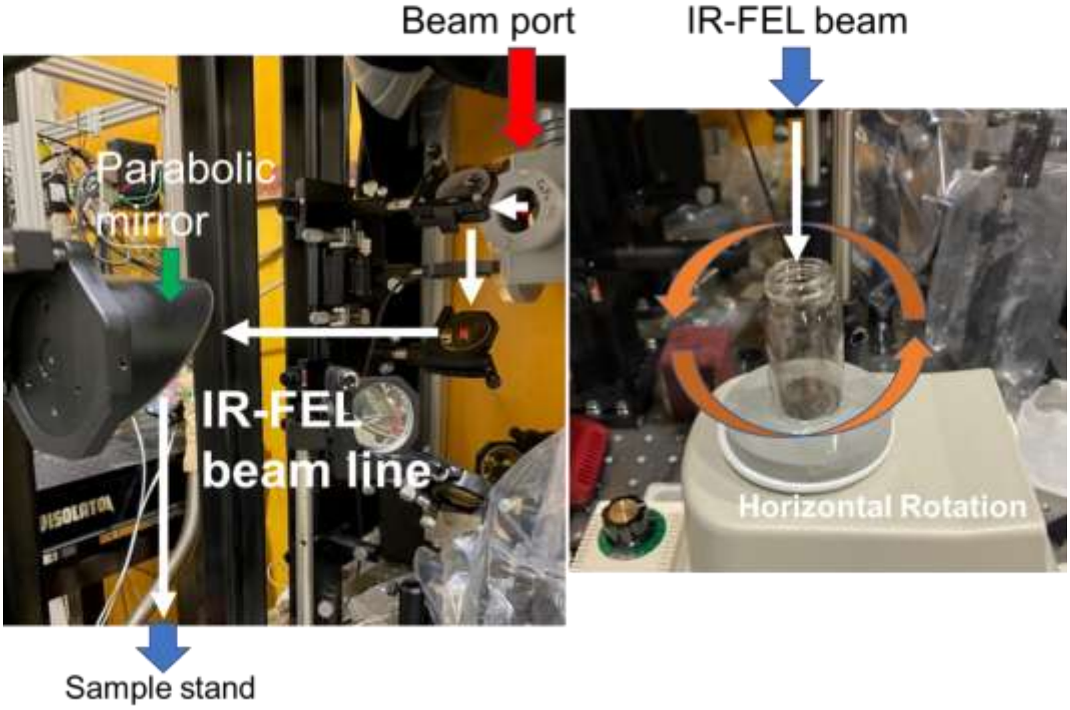

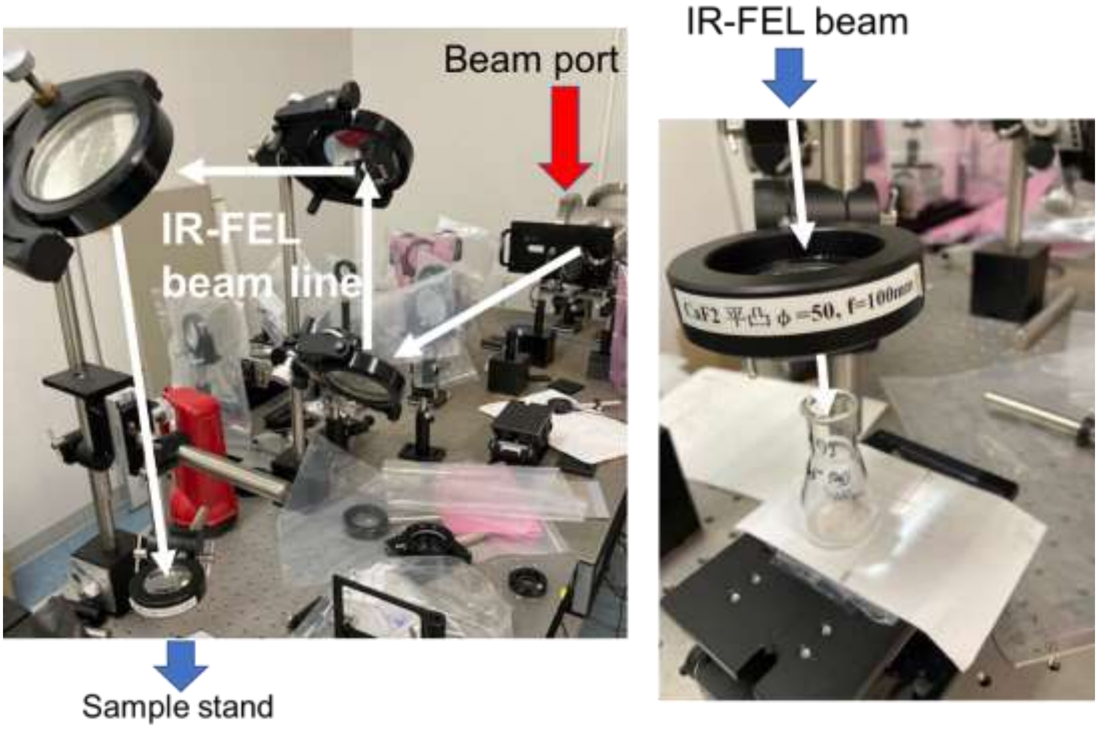

Figure S2. Experimental setup. Upper: mid-IR FEL irradiation system; below: near-IR FEL irradiation system.
